# Testing of rapid evaporative mass spectrometry for histological tissue classification and molecular diagnostics in a multi-site study

**DOI:** 10.1038/s41416-024-02739-y

**Published:** 2024-09-18

**Authors:** Martin Kaufmann, Pierre-Maxence Vaysse, Adele Savage, Loes F. S. Kooreman, Natasja Janssen, Sonal Varma, Kevin Yi Mi Ren, Shaila Merchant, Cecil Jay Engel, Steven W. M. Olde Damink, Marjolein L. Smidt, Sami Shousha, Hemali Chauhan, Evdoxia Karali, Emine Kazanc, George Poulogiannis, Gabor Fichtinger, Boglárka Tauber, Daniel R. Leff, Steven D. Pringle, John F. Rudan, Ron M. A. Heeren, Tiffany Porta Siegel, Zoltán Takáts, Júlia Balog

**Affiliations:** 1 Department of Surgery, Queen’s University, Kingston, ON Canada; 2 Gastrointestinal Diseases Research Unit, Kingston Health Sciences Centre, Kingston, ON Canada; 3 Maastricht MultiModal Molecular Imaging (M4I) Institute, Division of Imaging Mass Spectrometry, Maastricht University, Maastricht, NL Netherlands; 4 Department of Surgery, Maastricht University Medical Center + (MUMC+), Maastricht, NL Netherlands; 5 Department of Otorhinolaryngology, Head & Neck Surgery, MUMC+, Maastricht, NL Netherlands; 6 Division of Systems Medicine, Department of Metabolism, Digestion and Reproduction, Imperial College London, London, UK; 7 Department of Pathology, MUMC+, Maastricht, NL Netherlands; 8 GROW School for Oncology and Reproduction, MUMC+, Maastricht, NL Netherlands; 9 School of Computing, Queen’s University, Kingston, ON Canada; 10 Department of Pathology, Queen’s University, Kingston, ON Canada; 11 Department of General, Visceral and Transplantation Surgery, RWTH University Hospital Aachen, Aachen, Germany; 12 NUTRIM School of Nutrition and Translational Research in Metabolism Faculty of Health, Maastricht University, Maastricht, NL Netherlands; 13 Imperial NHS Trust, London, UK; 14 Signalling and Cancer Metabolism Team, The Institute of Cancer Research, London, UK; 15 Qamcom Research & Technology Central Europe, Budapest, Hungary; 16 Department of Surgery and Cancer, Biosurgery and Surgical Technology, Imperial College London, London, UK; 17 Waters Corporation, Wilmslow, UK; 18 Waters Research Center, Budapest, Hungary

**Keywords:** Lipidomics, Diagnostic markers, Mass spectrometry

## Abstract

**Background:**

While REIMS technology has successfully been demonstrated for the histological identification of ex-vivo breast tumor tissues, questions regarding the robustness of the approach and the possibility of tumor molecular diagnostics still remain unanswered. In the current study, we set out to determine whether it is possible to acquire cross-comparable REIMS datasets at multiple sites for the identification of breast tumors and subtypes.

**Methods:**

A consortium of four sites with three of them having access to fresh surgical tissue samples performed tissue analysis using identical REIMS setups and protocols. Overall, 21 breast cancer specimens containing pathology-validated tumor and adipose tissues were analyzed and results were compared using uni- and multivariate statistics on normal, WT and PIK3CA mutant ductal carcinomas.

**Results:**

Statistical analysis of data from standards showed significant differences between sites and individual users. However, the multivariate classification models created from breast cancer data elicited 97.1% and 98.6% correct classification for leave-one-site-out and leave-one-patient-out cross validation. Molecular subtypes represented by PIK3CA mutation gave consistent results across sites.

**Conclusions:**

The results clearly demonstrate the feasibility of creating and using global classification models for a REIMS-based margin assessment tool, supporting the clinical translatability of the approach.

## Introduction

Rapid evaporative ionisation mass spectrometry (REIMS) is the functional combination of electrosurgery and on-line mass spectrometric analysis of surgical aerosols [1–3]. Accurate differentiation of cancer versus non-cancerous tissue types by REIMS has been applied to breast [4], colon [5], ovarian [6] and cervical [7] pathologies, and suggests that REIMS could be a valuable tool to support intraoperative decision-making, with potential to improve patient outcomes. The goal of the oncology surgeon is to remove all cancer cells within a margin of non-cancerous tissue while minimizing the destruction of non-cancerous tissue. Margin status is most often determined postoperatively, sometimes occurring weeks after surgery. While pathologists examine tissue ex vivo at microscopic and cellular levels with up to <1 µm resolution by light microscopy, the surgeons must make intraoperative decisions based on macroscopic assessment in real-time, achieving a resolution of only ~1 mm when image guidance is employed [8]. Furthermore, tumour margins are not always uniform and can contain microscopic projections that emanate into surrounding tissues. As a result of these challenges, the surgeon can inadvertently encroach into the tumour bed, resulting in positive margins in 10–50% of procedures [9–11]. Positive margins can affect patient outcomes, potentially requiring further surgery and delays in adjuvant treatment. REIMS has the potential to address these challenges, as it could provide real-time, intraoperative classification of tissue at the metabolomics level, using pre-designed classification models based on mass spectral databases; with the ultimate goal of helping the surgeon avoid a positive margin before the patient leaves the operating theatre.

Implementation of REIMS for future diagnostic use faces potential challenges such as differences in site-specific electrosurgical units and tissue heterogeneity and handling, which could impact the accuracy of tissue classification models. Therefore, the analytical performance of REIMS, and the performance of multivariate models used for tissue classification requires rigorous investigation at a multi-site level. We previously created an international consortium of four sites in the United Kingdom, Europe and Canada with technically identical apparatus to evaluate the multi-site performance of REIMS using food-grade meats [12]. Importantly, we found that variability in REIMS spectra could be minimized by harmonizing instrument settings and tissue handling/sampling techniques, facilitated by establishing single source reference material.

**Fig. 1:**
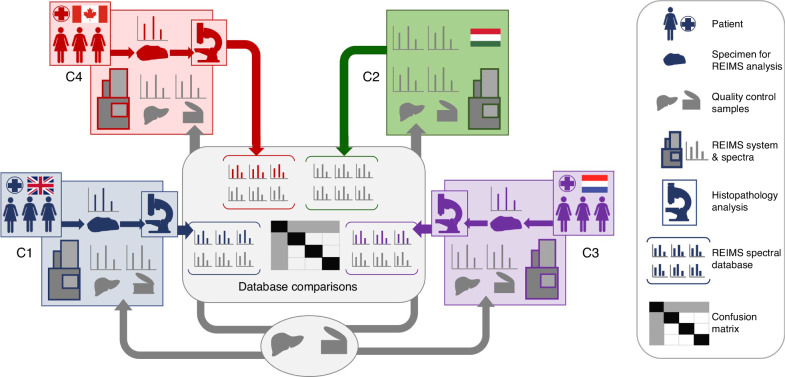
Workflow for multicenter study of clinical samples. Human breast tissue was obtained from patients who received surgical treatment for breast cancer at the following sites: Imperial College London (C1), Maastricht University (C3) and Queen’s University (C4). Tissue was analyzed by REIMS and validated by histopathologists. Quality control material including two batches of single-source pork liver and meat homogenate (National Institute of Standards and Technology (NIST), Standard reference material 1546a) was analyzed at all four centres including Waters Research Center (C2) to compare method performance.

In the current study, our objective was to evaluate the clinical applicability of REIMS in breast cancer surgery. We integrated our harmonized REIMS platform into surgical pathology workflows at three sites to study human breast tissue obtained from patients undergoing surgical treatment of invasive breast cancer. Breast tissue recognition models based on histopathology-validated mass spectra were created at each site, and the classification accuracy of those models was tested on data obtained at the other two sites (Fig. 1). Since fatty acids (FAs) ranked among the most significantly altered chemical species in breast cancer as compared with normal breast adipose, we then explored FA profiles across tumor tissue stratified by presence of *PIK3CA* mutation, which occurs in 40% of patients with hormone receptor-positive and human epidermal growth factor receptor 2 negative cancers [13, 14]; and shown to increase tissue arachidonic acid concentrations via PI3K signalling [15]. Our results reveal that REIMS is a robust tool that can identify invasive breast cancer across multiple sites in the United Kingdom, Europe and Canada; but also demonstrates the utility of REIMS as a semi-quantitative, targeted tool that can reveal mechanistic insights based on oncogene-induced metabolic biomarkers identified in breast cancer.

## Materials and methods

### Materials and reagents

Isopropanol and water (UPLC/MS grade) were purchased from Honeywell (VWR, NL). Leucine enkephalin (LeuEnk) was purchased from Sigma–Aldrich (St. Louis). Sodium hydroxide was purchased from Merck (Darmstadt, Germany).

### Samples and logistics

#### Non-clinical reference samples

Reference samples consisted of: (1) NIST reference meat homogenate (Standard reference material^Ⓡ^ 1546a, National Institute of Standards and Technology, NIST); and (2) two individual batches of pork liver procured by Imperial College London (Center 1 (C1), London, United Kingdom and shipped to three other participating centres comprising Waters Research Center, Budapest, Hungary (C2); Maastricht MultiModal Molecular Imaging Institute, Maastricht The Netherlands (C3); and Queen’s University, Kingston Canada (C4). Pork liver samples were shipped from C1 to the other sites on dry ice and stored in low-temperature (−80 °C) freezers until analysis (Fig. 1) as previously described [12].

#### Clinical samples

Human breast tissue was collected at three sites affiliated with clinical centers, from patients who underwent surgery for invasive breast cancer over 2018-2019 (Fig. 1). Informed consent was obtained, and study approval was granted as per the following local medical ethics committees: Imperial College London (C1); East of England—Cambridge East Research Ethics Committee, REC reference 14/EE/0024; Maastricht University (C3), medical ethics committee of Maastricht University Medical Center (MUMC+) permit No. METC 16-4-168; and Queen’s University (C4) Health Sciences Research Ethics Board, permit No. 6023032. After macroscopic examination, a site-specific breast pathologist (four pathologists in total) selected tumour and/or normal tissue samples from 6-8 breast cancer surgery cases from each site. Samples were stored in a low-temperature freezer (−80 °C) until REIMS analysis was performed.

### Sampling with electrosurgery

All tissues were allowed to reach room temperature before analysis and placed on a return electrode. If necessary, samples were wetted with deionised water. Electrosurgical dissection was carried out using commercial electrosurgical generators (C1: COVIDIEN Ltd. Triad, Ireland; C2: ERBE Elektromedizin GmbH ICC-350, Germany; C3/C4: COVIDIEN Ltd. Force Fx) providing power-controlled sinusoidal 330 kHz alternating current. Tissue was sampled using a custom diathermy pencil with a smoke evacuation line (Waters Research Center, Budapest Hungary). Each sampling event was conducted for 3–5 s.

#### Settings for the sampling of non-clinical samples

The generator was used in cut mode with an optimized power setting (i.e. 10 W for the NIST reference meat homogenate, 20 W for the pork liver samples). In order to maximize reproducibility, the diathermic knife was maintained in a semi-vertical position above the tissue during sampling. NIST and pork liver reference material was analyzed before and after each series of breast measurements, and used as quality control to assess instrument variability across sites. Measurements were performed two times per day, on two consecutive days for each sample. Each measurement consisted of 3–5 sampling points, typically lasting 4–8 s in duration.

#### Settings for the sampling of clinical samples

Previous work revealed lipid class-dependent differences in signal intensity in ‘cut’ as compared with ‘coagulation mode’ [4]. Thus, we analyzed all samples retrospectively using both modes where possible. In a clinical setting, we anticipate that this would enable continuous intraoperative tissue classification if the surgeon switches mode. Optimized generator power settings used, included 15–70 W in cut mode, or 10–30 W in coag. The blade on the diathermic knife was cut in half to maximize the aspiration of the plume into the mass spectrometer.

### REIMS – qTOF instrumentation

At each site, data was acquired on a Xevo^TM^ G2-XS quadrupole time-of-flight (qTOF) mass spectrometer fitted with a REIMS source (version III, research use only) (Waters, Wilmslow UK). Operating parameters were kept constant among sites, and instrument status was verified using a common checklist based on a harmonized REIMS protocol across all four sites [12]. Briefly, instruments were connected to a 7 bar gas (pressurized air or Nitrogen) supply. Time-of-flight (TOF) and backing pressures were in the range of <e^−7^ and 1.3 mbar, respectively. The smoke produced by electrocautery was aspirated via a Venturi pump connected to the REIMS interface. The heated coil in the REIMS source was kept at 8-900 °C. Data were acquired in “sensitivity” and negative ionization mode within the mass-to-charge *m/z* range of 100–1500. The mass resolution was above 15.000 full width at half-maximum. Instrument calibration was performed or checked with sodium formate before each measurement series. A solution of leucine-enkephalin at a concentration of 0.05 ng/µl (prepared in isopropanol) was continuously infused during acquisition at a flow rate of 150 µl/min for external lock mass correction. MS/MS fragmentation of molecular species was carried out using nitrogen as collision gas with 30–45 eV collision energy according to the exact mass and molecular species type.

### Histopathology examination of clinical samples

After REIMS analysis, the remaining tissues were fixed in formalin (Unifix, Klinipath) and embedded in paraffin. Tissue sections were then stained with Haematoxylin and Eosin (H&E) and examined by a breast pathologist. One or two pathologists at each of the three clinical centres (four pathologists in total) examined the tissues obtained and analyzed at their respective centres using standard-of-care methods. A representative tissue section from each specimen was assessed microscopically for the percentage of cancerous versus non-neoplastic tissue surrounding the area analyzed by REIMS, as annotated by the pathologists (Supplementary Figure S1). Spectra were assigned the label ‘breast cancer’ if the region surrounding the sampling point contained at least 30% breast cancer. Spectra containing < 30% breast cancer were excluded. Spectra containing only 100% normal breast adipose surrounding the sampling point, were labelled as normal breast tissue. Spectra containing < 30% breast cancer were excluded. We adopted a ‘30% cancer’ criteria to balance the number of spectra available in the breast cancer class with the risk of sampling/labelling error that can occur by extrapolation of class labels for spectra generated for ablated tissue, based on the tissue surrounding the REIMS sampling area.

### Data analysis

Mass spectral processing and multivariate data analysis were performed using the Abstract Model Builder (AMX) software ([beta] version 1.0.1581.0, Waters Research Center). All mass spectra were processed as follows: (i) background subtracted; (ii) mass shift corrected against the reference peak of deprotonated LeuEnk [M−H]^−^ at *m/z* 554.26; (iii) binned to 0.1 Da (within the mass range *m/z* 600–1000—corresponding to the region of abundant phospholipids and triglycerides); iv) normalized against the total ion count (TIC). Multivariate analysis was based on principal component analysis/linear discriminant analysis (PCA/LDA). PCA was performed with a maximum of *n* = 25 dimensions and LDA with *n*-1 dimensions where *n* corresponds to the number of variables introduced in the model. Cross-validation tests were performed by building the site-specific classifiers to recognize the data generated on the other sites. Data points were marked as outliers if they deviated 5 x standard deviation (SD). Inter- and intra-site and user variability was assessed using cosine similarity measure and KNN-classifier and all relevant site-specific peaks were listed using HSIC Lasso using Python 3.10. To identify specific peaks differentially abundant in normal and cancerous tissue, we used a Support Vector Machine (SVM) based algorithm in Python 3.7 using sklearn.svm.SVC function with l1 norm and a parameter of *C* = 500 to select features. To explore the reproducibility of FA signatures in breast cancer tissue stratified by *PIK3CA* mutational status, we used a MicrobeLynx model (Waters) [16] based on selected FA peaks: *m/z* 279.23, 307.26, 305.25, 303.23, 331.26, 329.25; normalized to the sum of FA peaks. Briefly, a channel was created from each FA peak using the median and standard deviation of each class, and the likelihood of a novel sample being part of each channel’s distribution was calculated. The sample was classified into the class with the highest likelihood. Five-fold cross-validation was used to assess accuracy. The relative abundance of selected ions-of-interest is presented as box-and-whisker plots using the method of Tukey (Graphpad PRISM). Boxes depict the median and span the 25–75th percentile. Whiskers extend to the highest and lowest data point if ≤ the 75th percentile plus 1.5× the interquartile range (IQR), or ≥ the 25th percentile minus the IQR. Individual data points are not shown. Alternatively, data points are shown if > the 75th percentile plus 1.5× IQR or <25th percentile minus 1.5× IQR. In this case, the whiskers extend to the 75th percentile plus 1.5× IQR and the 25th percentile minus 1.5× IQR. Two-tailed *t*-tests (unpaired) were used to assess differences in the abundance of normally distributed *m/z* bins, ratios, or cosine similarity scores, where significance was indicated by *p* < 0.05. Welch’s correction was applied when comparing data with unequal variances. Code availability: All codes used in this study are available, with restrictions, by contacting the corresponding author.

### *PIK3CA* mutation analysis

A PNA-Clamp *PIK3CA* Mutation Detection Kit (Panagene, PNAC-4001), was used to detect *PIK3CA* mutations in primary breast tumor samples, according to the manufacturer’s instructions. Briefly, DNA was extracted from 10 consecutive sections of 10 μm thickness tissue from FFPE blocks using the QIAamp DNA FFPE tissue kit (QIAGEN, 56404). Mutations in exon 9 (helical domain) and exon 20 (kinase domain) of *PIK3CA* were assessed. More specifically, reactions were performed using 10 ng DNA with a SYBR Green PCR reaction premix and primer premixes detecting E542, E545, Q546 and H1047 mutations using the TProfessional Thermocycler (Analytik Jena Biometra). The following cycle reactions were used: pre-denaturation for 5 min at 94 °C, followed by 40 cycles of 30 s at 94 °C (denaturation), 20 s at 70 °C (peptide nucleic acid clamping), 30 s at 63 °C (annealing) and 30 s at 72 °C (extension).

## Results

### Quality control of REIMS methodology using single-source pork liver and NIST reference material

**Fig. 2:**
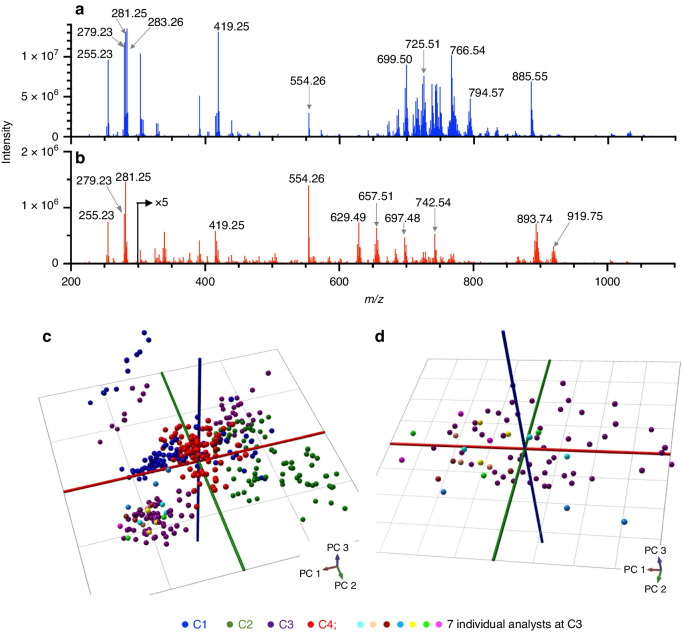
Multi-site characterization of quality control material. Representative REIMS spectra from two quality control materials analyzed: **a** single-source pork liver and **b** NIST meat homogenate. PCA plots of pork liver quality control data acquired at all sites **c** previously-acquired pork liver sampling points analyzed at C3 by 7 analysts are included in comparison to all quality control data acquired across sites in (**c**) and in comparison to pork liver quality control data acquired only at C3 (**d**).

Single-source pork liver and NIST meat homogenate served as quality control samples, analyzed by REIMS alongside clinical tissue samples at each centre using identical instrument configurations. Representative REIMS spectra from pork liver and NIST meat homogenate are shown in Fig. 2a, b. We observed differences in signal intensity, and distinct relative abundance of lipids including *m/z* 885.55 [PI(38:4)−H]^−^, 893.74 [TG(52:2)+Cl]^−^, and 629.49 [DG(34:1)+Cl]^−^, between the two quality control materials. Accordingly, multivariate analysis based on supervised PCA/LDA successfully separated the liver versus NIST samples and revealed little site-dependent clustering (not shown). To evaluate the variability of spectra based on possible centre-dependent biases, we plotted only the pork liver data using unsupervised PCA in Fig. 2c. Some centre-dependent biases could be discerned in the data, however, cross-validation revealed that the correct site could be identified only 50% of the time based on PCA/LDA suggesting that centre-dependent bias was minimal. Furthermore, when NIST meat homogenate was included in the model, the two quality control materials were always correctly classified during cross-validation across all centres. Using a KNN classifier with cosine distance, we found that pork liver spectra could be accurately identified by the site (Accuracy = 94–98%). However, it was mainly *m/z* bins corresponding to low-abundance peaks and chemical noise that appeared to drive the site-based separation of the pork liver spectra, and not the major characteristic tissue peaks (Supplementary Figs. S2 and S3). We previously demonstrated that slight differences in sampling technique by analysts from different sites were minimal, when all seven analysts from across each centre conducted multiple REIMS analyzes of pork liver using the same instrument at C3 [12]. These data points are included in Figure 2c for reference, and grouped with the pork liver sampling points completed by C3 for quality control purposes in the current study. The plot in Fig. 2d shows only the pork liver data points from C3, alongside the previous data acquired by different analysts at C3. Figure 2d reveals that the analyst-dependent variability in spectra was no greater than the spectral variability associated with multiple sampling points conducted by C3’s designated analyst. Spectral similarity across analysts was also demonstrated by high cosine similarities of 0.97–0.99, comparing spectra acquired by each analyst to C3’s quality control data. Cosine similarity values were not significantly different among analysts (Supplementary Fig. S4).

#### Characterization of human breast tissue by REIMS across sites

**Fig. 3:**
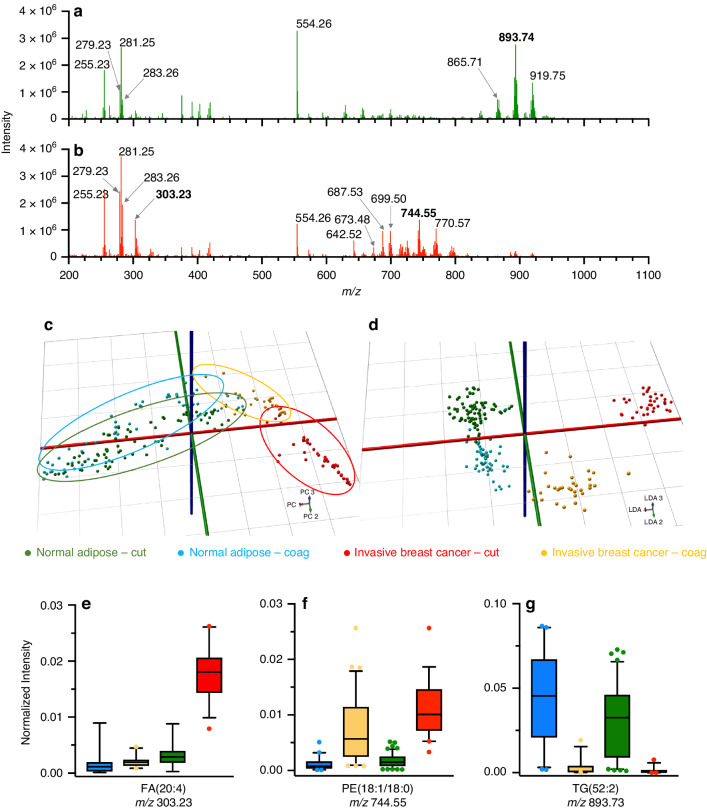
Multi-site characterization of human breast tissue. Representative REIMS spectra from either normal breast adipose **a** or invasive breast cancer **b**. The spectra shown are 1 s scans acquired using cut mode, and subjected to background subtraction and lockmass correction. The PCA **c** and PCA/LDA **d** plots compare the overall tissue-type variability between cut and coagulation modes. **e–g** Differential abundance of selected ions identified by peak picking, used to create a model with only 11 targeted ions.

We analyzed human breast samples collected from patients undergoing breast cancer surgery at our three sites affiliated with clinical centres (C1, C3, and C4). Each site obtained invasive breast cancer and/or normal breast samples from specimens resected from 6–8 patients and sampled them by REIMS in cut and coagulation mode, from which a total of 260 spectra were included (Table 1). We included invasive breast cancer spectra containing at least 30% tumour cells around the sampling site, as well as normal spectra that were free of tumour cells based on histopathology analysis as described in Materials and Methods. Spectra were excluded if there was a discrepancy between the gross tissue label and the histopathology-assigned label, or if the intensity of the scan was low (i.e. below 2 × 10^4^). Representative spectra from either normal breast adipose or invasive breast cancer acquired in cut mode are shown in Fig. 3a, b. Representative spectra from all sites for both cautery modes are shown in Supplementary Figs. S5 and S6. Not surprisingly, the intensity of the REIMS signals differed greatly among the sites, and the signal-to-noise ratio of spectra obtained from the human breast was significantly lower compared to the pork liver and NIST reference material, owing to the smaller-sized samples collected from the surgery. A remarkably consistent pattern of *m/z* ratios in adipose as compared with invasive breast cancer was observed among all sites. For example, triglycerides (*m/z* 850–1000) dominated the spectra acquired from normal tissue, but were mostly absent from tumour spectra, which exhibited a relatively high abundance of phospholipids, such as *m/z* 699.50 50 [PE(34:1)−NH_4_]^−^/[PA(36:2)−H]^−^ and 744.55 PE[(18:1/18:0)−H]^−^, in agreement with a previous study [4]. Highly abundant triglycerides observed in normal breast include *m/z* 865.71 [TG(50:2)+Cl]^−^, 893.74 [TG(52:2)+Cl]^−^ and 919.75 [TG(54:3)+Cl]^−^.

**Table 1:** Breast cancer patient demographics and sampling points.

Center	Patient	Diagnosis^a^	Age	Hormone receptor status^b^			*PIK3CA* mutation status^c^	No. burns (*N*)^d^		No. burns (*T*)^d^	
				ER	PR	Her2		CT	CG	CT	CG
C1	1	ILC	54	+	+	n/d^e^	n/d	5	4	4	2
	2	IDC	40	+	+	+	E542R/E545X/Q546X	3	2	0	0
	3	IDC	76	+	+	−	E545X/Q546X	5	5	0	0
	4	IDC	47	+	+	+	n/d	0	0	0	3
	5	IDC	81	+	+	−	n/d	4	5	0	4
	6	DCIS	68	n/d^d^	n/d	n/d	H1047R	4	3	0	0
C3	1	IDC	43	+	+	−	WT	5	1	5	3
	2	IDC	73	+	+	−	E545X/Q546X/H1047R	5	2	2	0
	3	ILC	54	+	−	−	WT	5	4	1	0
	4	ILC	86	+	+	n/d	WT	5	3	3	4
	5	IDC	71	+	+	−	WT	5	3	5	5
	6	ILC	49	+	+	−	E545X/Q546X	0	2	3	0
	7	IDC	62	+	+	+	E545X/Q546X/H1047R	0	0	3	3
	8	IDC	80	−	−	−	E542R/H1047R	5	4	2	5
C4	1	IDC	84	−	−	n/d	E542R/C420R/H1047R	8	6	2	2
	2	IDC	70	+	+	−	WT	8	4	0	0
	3	IDC	56	+	+	−	WT	9	5	0	0
	4	IDC	69	+	+	−	n/d	3	2	3	0
	5	IDC	57	−	−	−	E545X/Q546X	0	0	3	1
	6	IDC	88	−	−	+	H1047R	0	0	3	0
	7	IDC	79	+	+	−	WT	3	2	0	0
Total	21	*n/a*^f^						82	57	39	32

^a^*ILC* invasive lobular carcinoma, *IDC* invasive ductal carcinoma, *DCIS* ductal carcinoma in situ.

^b^*ER* estrogen receptor, *PR* progesterone receptor, *Her2* human epidermal growth factor receptor receptor 2.

^c^*PIK3CA* mutational status associated with tumor tissue sampled. *WT* wildtype.

^d^Number of sampling events in either normal (N) or tumor (T) tissue, using cut (C) or coagulation (CG) mode.

^e^Not determined.

^f^Not applicable.

Intra-site and inter-site variability in spectra was studied by using the cosine similarity metric (Supplementary Figs. S7 and S8). Most intra-site cosine similarities were > 0.9, with cancer spectra exhibiting somewhat lower similarity scores than normal adipose, most notably in coagulation mode. Inter-site cosine similarity measures were typically lower than intra-site, suggesting the presence of inherent intra-site variability. As observed with the intra-site measures, cosine similarity was typically lower for cancer tissue, particularly in coagulation mode. While not surprising that cancer tissue exhibited greater variability than normal, these data suggest that overall sample-based variability is relatively low, as compared with site-based variability, and that ablation of cancer tissue in coagulation mode led to the lowest median inter-site similarity score of 70%. The majority of significant *m/z* bins that contribute to inter-site variation in spectra appear to be low-abundance bins related to noise, from which a specific, measured *m/z* cannot be discerned. Exceptions include major triglycerides *m/z* 891.72, 865.71 and 895.75 that contribute to site-based differences in normal tissue (both cut and coagulation). Interestingly, the peaks contributing to site-based variability in breast cancer spectra include residual triglycerides (*m/z* 925.72, 923.78) as well as a glycerophospholipid (*m/z* 742.54) (Supplementary Fig. S7). The volcano plot analysis in Supplementary Fig. S9 indicates the number of *m/z* bins that differ significantly in either mode, in normal adipose or breast cancer. As many of the bins correspond to low-abundance peaks or noise, peaks with the most significant PCA loading scores were also considered. While certain abundant triglycerides were distinctly elevated in the coagulation mode (*m/z* 909.77, 925.72), the most abundant species (*m/z* 893.74, 891.72 or 919.75) did not differ significantly between the two modes. In breast cancer tissue, abundant *m/z* bins with the most significant loadings that differentiate cut and coagulation were shown to be statistically significant as well. These data suggest that electrosurgery mode impacts the spectral fingerprint of breast cancer tissue more so than that of normal adipose tissue, as indicated in the representative spectra shown in Supplementary Figures S5 and S6.

#### Multivariate analysis of human breast spectra and multi-site tissue classification

A total of *n* = 210 pathology-annotated spectra were included in the multivariate model; including *n* = 71 invasive breast cancer (39 cut/32 coag), and *n* = 139 normal (82 cut/57 coag). Figure 3 shows PCA (3C) and PCA/LDA plots (3D) where the separation between spectra labeled as cancerous and normal is almost complete, independent of the site. We observed a correct classification rate of 96.4 (range 92–100%) when creating a model with one site’s data and testing it against data from the remaining two sites (Table 2). To see if a site-diverse training set could improve tissue classification accuracy, we built a model where the training set contained the data of 2 sites and classified the data acquired at the third site; all sites were left out once (cross-validation by site). A 97.11% correct classification rate with 1.4% false negative and 5% false positive rates was observed (Table 3). We also performed ‘leave-one-patient-out’ cross-validation by keeping all data in the training set with the exception of data from one patient; resulting in a 98.57% correct classification rate with a 2.8% false negative and 0.72% false positive rate (Table 3). Supplementary Figure S10 depicts two spectra from C3 obtained in coagulation mode, that were misclassified by a model created using C4’s data. False negatives (Fig. S10A) and false positives (Fig. S10B) appear to be caused by low spectral quality, and the presence of glycerophospholipid peaks in scans labeled as normal. A total of 46 sampling points were initially excluded from model creation and testing that were determined to contain tumor cell content of < 30%, to minimize the impact of the spectral labelling error on the assessment of multisite performance. Upon classification of these points, 67.4% were correctly identified as ‘cancer’; or 73.8% when excluding 4 outliers. When testing individual scans within each of the 46 points, we found that 78.2% of the points that contained at least one scan classified as ‘cancer’ were correctly recognized. None of the individual scans tested were determined to be outliers.

**Table 2:** Correct classification rate for human breast tissue using models based on one site’s data.

Model used for training	Correct classification rate (%)	Rate of false negatives (%)	Rate of false positives (%)
C1	98.0	1.9	3.0
C3	100.0	0	0
C4	91.8	21.1	0

**Table 3:** Confusion matrix for classification of spectra from human breast based on one-site-out, or one-patient-out cross-validation.

True class ↓	Predicted class (no. of spectra)			Correct classification rate (%)	Rate of false negatives (%)	Rate of false positives (%)
	Tumor	Normal	Total			
Tumor	70^a^	1^a^	71	97.1	1.4	5.0
Normal	7^a^	132^a^	139			
Total	77	133	210			
Tumor	69^b^	2^b^	71	98.6	2.8	0.7
Normal	1^b^	138^b^	139			
Tota**l**	70	140	210			

^a^Predicted class based on leave one-site-out cross-validation.

^b^Predicted class based on leave one-patient-out cross-validation.

We subjected the same set of spectra with >30% cancer cell content to a feature selection algorithm, over the entire mass range. In total, 11 features were identified, 6 of which were relatively abundant in normal breast including four triglyceride and two fatty acid species. Five features that were relatively abundant in invasive breast cancer included PEs and FAs summarized in (Table 4) as well as two unidentified peaks: *m/z* 137.03 and *m/z* 134.04. A model based only on these 11 features resulted in the same classification rate as achieved using the *m/z* 600–1000 mass range –98.57%, with 4.23% false negatives and no false positives. The relative abundance of selected species is shown as boxplots in Figs. 3e–g.

**Table 4:** Characterization of ions abundant in invasive breast cancer and normal adipose

Abundant in	*m/z* (bin)	*m/z* (measured)	*m/z* (theoretical)	*m/z* (error, ppm)	Putative ID	Ion
Cancer	279.25	279.234	279.2329	3.9	FA(18:2)	[M−H]^−^
Normal	281.25	281.248	281.2486	2.1	FA(18:1)	[M−H]^−^
Cancer	303.25	303.233	303.2329	0.3	FA(20:4)	[M−H]^−^
Normal	629.45	629.488	629.4917	5.9	DG(18:1/16:0)	[M+Cl]^−^
Cancer	744.55	744.555	744.5549	0.1	PE(18:1/18:0) and PE-NMe2(18:1/16:0)	[M−H]^−^
Normal	865.75	865.710	865.7057	4.9	TG(50:2)	[M+Cl]^−^
Normal	893.75	893.733	893.7370	4.4	TG(52:2)	[M+Cl]^−^
Normal	919.75	919.748	919.7527	5.5	TG(54:3)	[M+Cl]^−^
Normal	925.75	925.725	925.7291	4.4	TG(58:10)	[M−H]^−^

### Characterization of fatty acid profiles from REIMS spectra

**Fig. 4:**
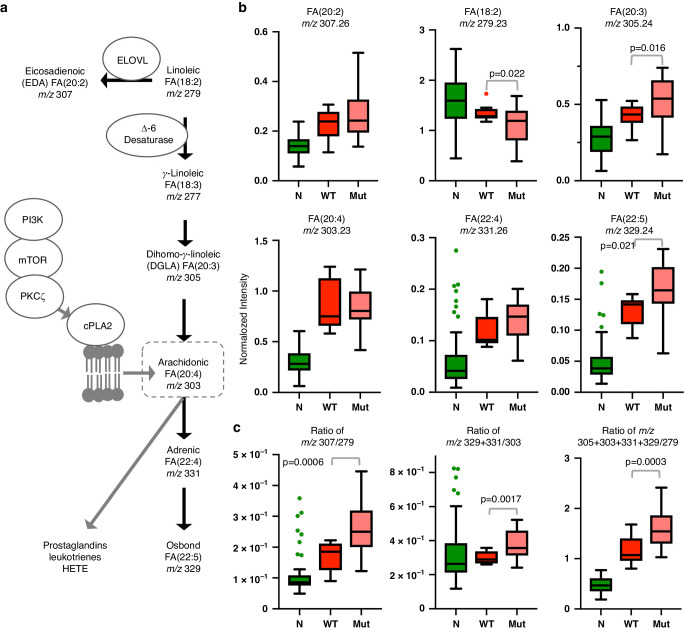
Fatty acid metabolism in breast tissue. Scheme of *ω*6 fatty acid metabolism **a** indicating selected enzymes involved, and the role of PI3K signaling in the liberation of arachidonic acid from the cell membrane by cPLA2. Fatty acids measured in the current study, emphasize pathways that contribute to tissue pools of arachidonic acid. Boxplots in **b** from a subset of data stratified by *PIK3CA* genotype indicate relative concentrations of the sum of selected fatty acids in normal breast adipose (*N*, *N* = 82) compared to tumor tissue harboring wildtype (WT, *N* = 12) or mutant (Mut, *N* = 23) *PIK3CA*. There was a significant difference in the level of all fatty acids between *N* and WT/Mut with all *p* < 0.005. **c** Boxplots of ratio analysis showing the change in the overall fatty acid metabolism.

Given that our feature-selection algorithm identified 3 out of 11 significant features as FAs (including arachidonic acid) as differentially abundant between breast tissue types (Table 4), we explored the relative abundance of all fatty acid peaks detectable by REIMS in cut mode. We detected 6 FAs, comprising a combination of saturated, and mono-/poly-unsaturated species many of which are associated with the *ω*6 fatty acid pathway (Fig. 4a) (Table S1). While [FA(18:1)-H]^−^ and [FA(18:2)-H]^−^ were more abundant in benign adipose than tumor tissue, all other downstream FAs including [FA(20:4)-H]^−^ (arachidonic acid) exhibited increased abundances in tumor tissue suggesting increased FA metabolism. Based on our previous work [15], we hypothesized that arachidonic acid would be elevated in breast cancer tissue harboring *PIK3CA* mutations. When spectra were stratified by *PIK3CA* mutation status, no increase in [FA(20:4)−H]^−^ was observed. However, the precursor [FA(18:2)−H]^−^ was decreased, whilst [FA(20:3)−H]^−^, [FA(22:4)−H]^−^ and [FA(22:5)−H]^−^ were increased in *PIK3CA-*mutated tumor tissue, suggesting enhanced arachidonic acid metabolism (Fig. 4a, b). The ratiometric analysis also suggested that the metabolism of arachidonic acid to other downstream FAs was increased in tissues harboring mutant compared to wildtype *PIK3CA* (Fig. 4c). Furthermore, increased metabolism of [FA(18:2)−H]^−^ to [FA(20:2)−H]^−^ was observed in mutant tissues. Given that PCA/LDA-based models would likely over-fit the limited number of sampling points available, we created a two-class MicrobeLynx model *(PIK3CA* wildtype vs. mutant) assuming Gaussian distribution for each of the 6 FA peak channels. 5-Fold cross-validation achieved an accuracy of 69.7% (sensitivity = 84.4%, specificity = 41.7%) pointing to factors other than *PIK3CA* genotype that could affect *ω*6 FA levels in breast tumor tissue. When we studied FA profiles in samples stratified by hormone receptor status, metabolism of [FA(18:2)−H]^−^ to [FA(20:2)−H]^−^ was enhanced in triple-negative as compared with estrogen receptor/progesterone receptor positive samples (ERPR+), Figure S11. While the metabolism of arachidonic acid did not differ significantly between triple-negative and ERPR+ tumor samples on the basis of individual FAs or ratios, overall metabolism of [FA(18:2)−H]^−^ through the *ω*6 pathway was enhanced in triple-negative samples, based on the sum of all [FA(18:2)−H]^−^ products relative to precursor [FA(18:2)−H]^−^, Figure S11.

## Discussion

Using harmonized REIMS methodology, we characterized normal breast adipose and invasive breast cancer samples obtained from patients who underwent standard-of-care surgical treatment for breast cancer involving local pathology assessment across three clinical sites. Differential abundance of ions in three classes of chemicals including phospholipids, triglycerides and fatty acids were found to accurately classify the two tissue types. On the basis of altered phospholipid-to-triglyceride, single-site models provided a correct classification rate with > 92%; and by combining two sites’ data into a single model and performing one-site-out cross-validation gave an accuracy of at least 97%. We conclude that the phospholipid/triglyceride *m/z* range is an informative signature for recognizing invasive breast cancer across sites, which is consistent with a larger single-site cohort study published by C1 in 2017 [4]. A more targeted model based on 11 selected ions across a broader *m/z* range that included fatty acids, achieved a correct classification rate of 98%. Although fatty acid profiles discerned from REIMS spectra were consistent with oncogenic signaling pathways including *PIK3CA* and hormone receptor status, a larger study will be needed to identify mass spectral patterns that can be used for accurate recognition of breast cancer subtypes across sites that may aid in post-operative clinical decision support [17]. Our results suggest that intraoperative characterization of breast cancer resection margins is possible. To improve the surgical utility of this tool, linking REIMS signals to preoperative and 3D intraoperative imaging will help guide the surgeon back to the precise location of a positive margin to resect more tissue [18–20]. The current study adds REIMS to a number of other imaging and ambient ionization techniques that have been evaluated at the multi-site level [21–24].

Upon evaluation of sources of spectral variability, spectra appeared to vary the least by the analyst, based on the analysis of pork liver quality control samples. Sample-based (intra-site) differences were also considered to be minimal, but variability in spectra increased when coagulation mode was used, particularly when breast cancer tissue was analyzed, which led to considerable variability when site-to-site comparisons were made. Methods used to identify the underlying site-dependent peaks pointed to low-abundance and/or noise-containing bins, however, triglycerides were the most frequently highlighted species contributing to site-based variability for which *m/z* ratios could be verified. Although this was not surprising for normal breast adipose tissue, triglycerides were also observed to contribute to spectral variability in breast cancer. Even though coagulation spectra exhibited the highest variability, none of the top bins were found to be selectively more abundant in coagulation mode. Spectral variability can be explained in part by the altered ohmic resistance of the different-sized samples collected from the surgery, tissue heterogeneity, and the challenging task of cauterizing adipose tissue with a monopolar device. We also note that certain causes of spectral variability are unique for tissues sampled ex vivo, and do not occur in spectra acquired intraoperatively. Upon examination of misclassified spectra, lower spectral quality and an unexpected balance of glycerophospholipids-to-triglycerides relative to the tissue label were observed. This could arise from a different tumor cell percentage in the ablated tissue relative to that extrapolated from the surrounding region by pathology (labeling error), and the presence of other non-cancerous epithelial cell types in normal tissue and/or tumor tissue. A limitation of REIMS continues to be the destructive nature of the technique, making the precise cell composition of sampling points challenging to confirm. Site-dependent biases in labeling error (concordance across pathologists) or clinical tissue harvesting were not controlled for, as our intention was to evaluate performance on independently acting, surgical-pathology workflows. However, this may have resulted in tissue misclassification depending on the site train/test combination; which we attempted to mitigate by 1) creating site-diverse models in addition to models from individual sites’ data, and 2) by including ‘breast cancer’ specimens only if >30% tumor cell content was determined by the pathologist. Encouragingly, 73.8% of the excluded tumor sampling points were correctly classified, but this suggests that the remaining 23.9% of the points contained tumor cell concentrations below a limit of detection that has yet to be determined or that sampled regions sparsely populated with tumor cells might not have contained any tumor cells. Each of these scenarios would potentially contribute to false negatives. Model creation and testing was focussed on the *m/z* 600–1000 region of the spectrum corresponding to structural/storage lipids and contains lower noise levels and fewer solvent interferences compared to lower mass rages. When included (*m/z* 100–1000), the lower mass range led to greater spectral variability. However, a targeted approach using the most differentially abundant ions between normal breast and breast cancer exhibited a superior tissue classification accuracy of 98%, in part by minimizing the impact of spectral variability on classification and by avoiding the inadvertent inclusion of noise-containing bins that occurs by including broad mass ranges. This observation points towards the feasibility of using triple quadrupole mass analyzers for iKnife applications in the future.

De novo lipid synthesis is a hallmark of tumorigenesis, which has prompted the interrogation of lipid metabolism pathways as both diagnostic and therapeutic targets in breast cancer. Under hypoxic conditions, increased production of fatty acids for use in the synthesis of cell membrane phospholipids is needed for rapidly proliferating cancer cells [25]. Current and previous REIMS studies demonstrate an increased abundance of phospholipid in comparison to normal breast tissue, an observation that is consistent with other mass spectrometry-based lipidomic techniques applied to fresh clinical samples [4, 26, 27]. This presumably occurs for multiple reasons, including the dysregulation of lipid metabolism in the cancerous tissue, as well as the large proportion of epithelial cells present in tumor relative to normal breast adipose [28]. Not surprisingly, triglycerides constitute the majority of the signal in spectra from normal breast as observed with other techniques [29]; since it is composed mainly of adipose tissue but also some epithelial cells associated with ducts and mammary glands that contribute to spectral variability. Our targeted approach identified increased levels of arachidonic acid in tumor tissues relative to normal, consistent with observations using other mass spectrometry-based methods [28, 30]. Arachidonic acid is released from phospholipid or triglyceride by phospholipase and gives rise to eicosanoids that promote tumor growth and inflammation [31]; a pathway enhanced in tumors possessing activation-of-function mutations in *PIK3CA* and dependent on a PI3K signaling cascade correlated with REIMS-detectable arachidonic acid (Fig. 4A) [15]. While we did not observe increased arachidonic acid levels in *PIK3CA* mutant tissues, we did however observe increased abundance of FA products downstream of arachidonic acid. A number of factors may explain why arachidonic acid did not appear significantly elevated in our *PIK3CA* mutant tissues including the broad range of heterogenous tumor cell content of our samples (30-100%) and the fact that tissue pools of arachidonic acid can also arise from delta-6-desaturation of linoleic acid (FA(18:2)) which is generally enhanced in breast cancer tissue but especially in estrogen receptor-negative samples [28]. These factors may rationalize why targeted models based on individual FAs achieved lower cross-validation accuracies when attempting to predict *PIK3CA* mutational status. The abundance of FAs did not differ when stratified by hormone receptor status, which suggests that *PIK3CA* genotype had a dominant effect on arachidonic acid metabolism. This was reinforced by FA ratios revealing that arachidonic acid metabolism was more prevalent in *PIK3CA-*stratified, but not receptor-stratified tissues, even though all tumor tissues exhibited increased overall FA(18:2) metabolism. Our small sample size, and static measurements in a heterogeneous tissue cohort may have resulted in confounding effects on FA metabolism that preclude mechanistic conclusions from our study. However, our results emphasize that REIMS signals are biologically relevant and align with metabolic aberrations known to occur in breast cancer tissue. This points to the utility of REIMS as a rapid tool to investigate oncogenic pathways, in addition to serving as an effective tissue profiling modality, which opens up the possibility of exploring the use of REIMS for patient stratification.

Taken together, REIMS can differentiate with high accuracy normal breast from invasive breast cancer tissues in clinical samples independently collected, analyzed and validated in the UK, Europe and Canada. We also show the possibility of mapping tumor subtypes based on the monitoring of fatty acid metabolism with REIMS. Our data and others show that models which maximize the number of data points used from the broadest cross-section of patients and regions possible will lead to a more accurate classification. Our results, in addition to the establishment of reference material and standard operating procedures, demonstrate that accurate intraoperative classification of breast tissue by REIMS is possible. All of our sites have developed clinical workflows for the use of mobile REIMS units in the operating theater, enabling us to test this hypothesis.

## Supplementary information

Supplementary

## Data Availability

Data is available within the manuscript and supplementary information.
