# Supplementary material for: Testing of rapid evaporative mass spectrometry for histological tissue classification and molecular diagnostics in a multi-site study
Source: Br J Cancer. 2024 Sep 18;131(8):1298–308. doi: 10.1038/s41416-024-02739-y (PMC11473823; doi:10.1038/s41416-024-02739-y)
Supplement: Supplementary file 1 — Supplementary [file 41416_2024_2739_MOESM1_ESM.pdf]

Supplementary Information

Testing of Rapid Evaporative Mass Spectrometry for Histological Tissue Classification and Molecular Diagnostics in a Multi-site Study

Martin Kaufmann,[1,2] Pierre-Maxence Vaysse,[3,4,5] Adele Savage,[6] Loes F.S. Kooreman,[7,8] Natasja Janssen,[9] Sonal Varma,[10] Kevin Yi Mi Ren[10], Shaila Merchant,[1] Cecil Jay Engel,[1] Steven W.M. Olde Damink,[4,11,12] Marjolein L. Smidt,[4,8] Sami Shousha,[13] Hemali Chauhan,[6] Evdoxia Karali,[14] Emine Kazanc,[14] George Poulogiannis,[14] Gabor Fichtinger,[9] Boglárka Tauber,[15] Daniel R. Leff, [16] Steven D. Pringle,[17] John F. Rudan,[1] Ron M.A. Heeren,[3] Tiffany Porta Siegel,[3] Zoltán Takáts,[6] Júlia Balog,[6,18]*

[1] Department of Surgery, Queen's University, Kingston, ON, Canada
[2] Gastrointestinal Diseases Research Unit, Kingston Health Sciences Centre, Kingston ON, Canada
[3] Maastricht MultiModal Molecular Imaging (M4I) Institute, Division of Imaging Mass Spectrometry, Maastricht University, Maastricht, NL
[4] Department of Surgery, Maastricht University Medical Center + (MUMC+), NL
[5] Department of Otorhinolaryngology, Head & Neck Surgery, MUMC+, NL
[6] Division of Systems Medicine, Department of Metabolism, Digestion and Reproduction, Imperial College London, London, UK
[7] Department of Pathology, MUMC+, NL
[8] GROW School for Oncology and Reproduction, MUMC+, NL
[9] School of Computing, Queen's University, Kingston ON, Canada
[10] Department of Pathology, Queen's University, Kingston ON, Canada
[11] Department of General, Visceral and Transplantation Surgery, RWTH University Hospital Aachen, Aachen, Germany
[12] NUTRIM School of Nutrition and Translational Research in Metabolism Faculty of Health, Maastricht University, NL
[13] Imperial NHS Trust, London, UK
[14] Signalling and Cancer Metabolism Team, The Institute of Cancer Research, London, UK
[15] Qamcom Research & Technology Central Europe, Budapest, Hungary
[16] Department of Surgery and Cancer, Biosurgery and Surgical Technology, Imperial College London, London, UK
[17] Waters Corporation, Wilmslow, UK
[18] Waters Research Center, Budapest, Hungary

* Corresponding author: Júlia Balog, PhD. Waters Research Center H-1031 Budapest, HU, +3630203352, Julia_balog@waters.com

Contents:

Figure S1: Histopathology annotation of specimens analyzed by REIMS 3

Figure S2: Classification of pork liver spectra by site 4

Figure S3: Chemical contamination in low-abundance *m/z* bins 5

Contents (continued):

Figure S4: Similarity of pork liver spectra among analysts 6

Figure S5: REIMS spectra from human breast tissue samples (cut) 7

Figure S6: REIMS spectra from human breast tissue (coagulation) 8

Figure S7: Site-based comparison of spectra from clinical samples (*m/z* 600-1000) 9

Figure S8: Site-based comparison of spectra from clinical samples (*m/z* 100-1000) 10

Figure S9: Differentially-abundant ions in cut as compared with coagulation mode 11

Figure S10: Examples of misclassified spectra 12

Table S1: Characterization of selected fatty acids in normal adipose and invasive breast cancer stratified by *PIK3CA* mutation and hormone receptor status 13

Figure S11: Fatty acid metabolism in breast tissue stratified by hormone receptor status 14

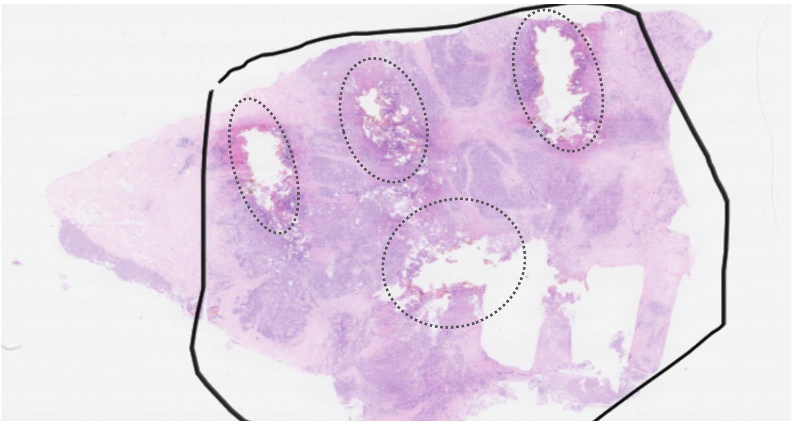

Supplementary Figure S1: Histopathology annotation of specimens analyzed by REIMS. Representative H&E micrograph of a human breast specimen analyzed by REIMS at C1. The solid line indicates the overall area determined to contain 90% invasive breast cancer. The areas encircled by dotted lines, indicate 4 specific regions sampled by REIMS in a single specimen.

A

true label \ predicted label	C1	C2	C4	C2
C1	99	0	0	0
C2	1	108	0	3
C3	1	0	89	0
C4	1	1	0	78

m/z (bin)[1]	*m/z* measured	Rank[2]
979.75	979.73	205
740.55	740.52	67
982.75	982.76	217
730.55	730.54	222
960.65	NA	607
835.55	835.56	84
847.55	847.56	139
780.55	780.56	176
934.65	NA	541
943.65	NA	602

B

true label \ predicted label	C1	C3	C4	C2
C1	90	2	6	1
C3	0	108	0	4
C4	0	1	89	0
C2	1	7	0	72

m/z (bin)[1]	*m/z* measured	Rank[3]
811.45	NA	397
512.45	512.47	97
780.55	780.56	246
756.55	756.56	387
730.55	730.54	327
746.55	746.55	130
661.45	661.49	482
716.55	716.52	77
689.55	689.52	342
957.75	957.56	1005

[1] Most abundant *m/z* bin from each of 10 groups of correlated bins selected by HSIC LASSO.
[2] Rank (bin number) based on relative abundance over 5000 bins (*m/z* 600-1000).
[3] Rank (bin number) based on relative abundance over 9000 bins (*m/z* 100-1000).

Supplementary Figure S2: Classification of pork liver spectra by site
REIMS spectra from pork liver quality control material could be accurately identified by site using a KNN classifier with cosine distance. Cross-validation by site is shown for both the *m/z* 600-1000 mass range (98% accuracy) (A) or the *m/z* 100-1000 mass range (94% accuracy) (B). *M/z* bins exhibiting the greatest site-based difference are shown, along with their rank by abundance.

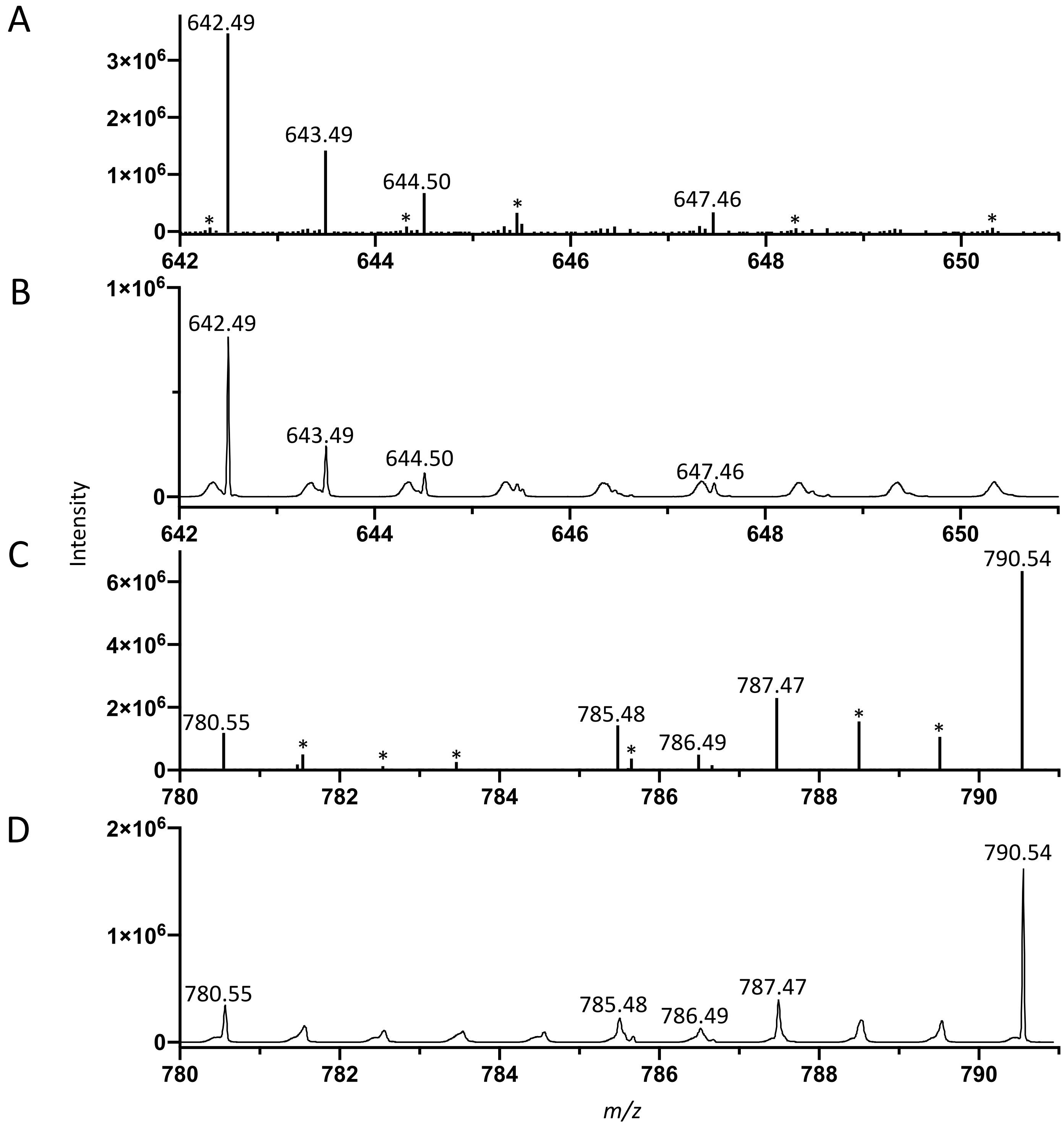

Supplementary Figure S3: Chemical contamination in low-abundance *m/z* bins.
Representative continuum and centroided spectra depicting specific *m/z* ranges from pork liver (A,B *m/z* 642-651) and (C,D *m/z* 780-791). Labeled peaks include low-abundance *m/z* ratios sufficiently resolved from chemical noise. However, peaks denoted with (*) are insufficiently resolved from, or contain mostly chemical noise. Models/ selected features that are able to classify spectra by site may be over-fitted to certain *m/z* bins containing low abundance peaks and/or chemical noise.

A

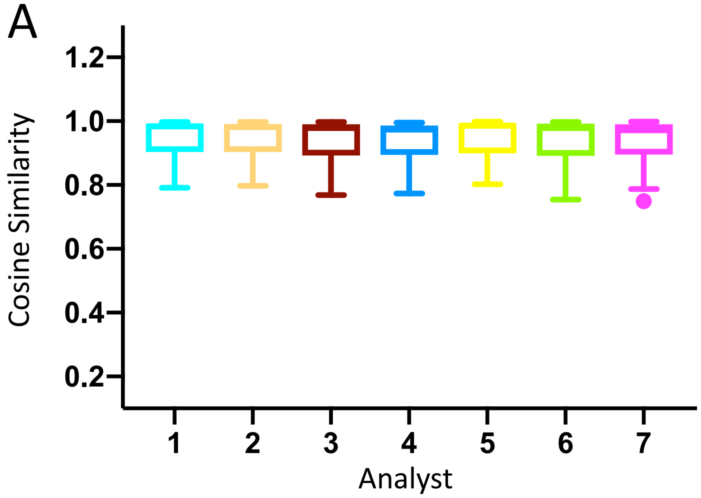

B

Analysts compared	*p* (*m/z* 600-1000)[1]	Significance[2]
1/2	0.9715	NS
1/3	0.1878	NS
1/4	0.1415	NS
1/5	0.8957	NS
1/6	0.1433	NS
1/7	0.0933	NS
2/3	0.1911	NS
2/4	0.1432	NS
2/5	0.8657	NS
2/6	0.1453	NS
2/7	0.0939	NS
3/4	0.9250	NS
3/5	0.1513	NS
3/6	0.8766	NS
3/7	0.7254	NS
4/5	0.1115	NS
4/6	0.9458	NS
4/7	0.7866	NS
5/6	0.1143	NS
5/7	0.0729	NS
6/7	0.8472	NS

[1] *p*-value for cosine similarity difference between analyst pairs over the *m/z* 600-1000 range.
[2] Significance denoted by: NS, not significant (p>0.05)

Supplementary Figure S4: Similarity of pork liver spectra among analysts.
Cosine similarity assessment of pork liver spectra acquired by seven analysts at C3, as compared with the C3's quality control spectra (A). Differences in cosine similarity values between analysts were not significant (B).

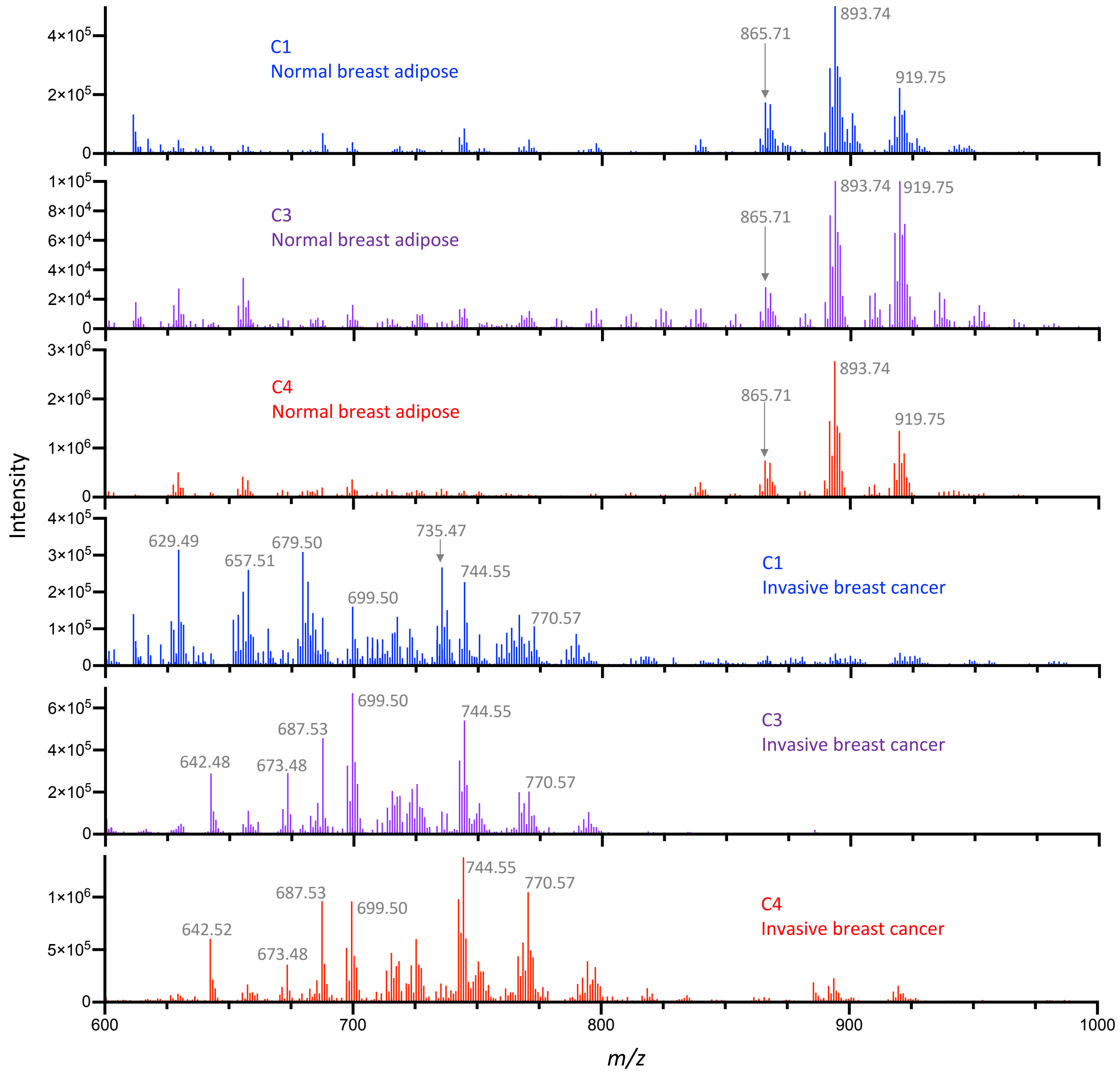

Supplementary Figure S5: REIMS spectra from human breast tissue samples (cut). Representative REIMS spectra acquired in cut mode from either normal adipose or invasive breast cancer samples acquired from patients being treated at the 3 Consortium sites affiliated with clinical centers. The spectra shown are 1s scans acquired using cut mode, subjected to background subtraction and lockmass correction. The tissue type was determined by a breast pathologist at each site.

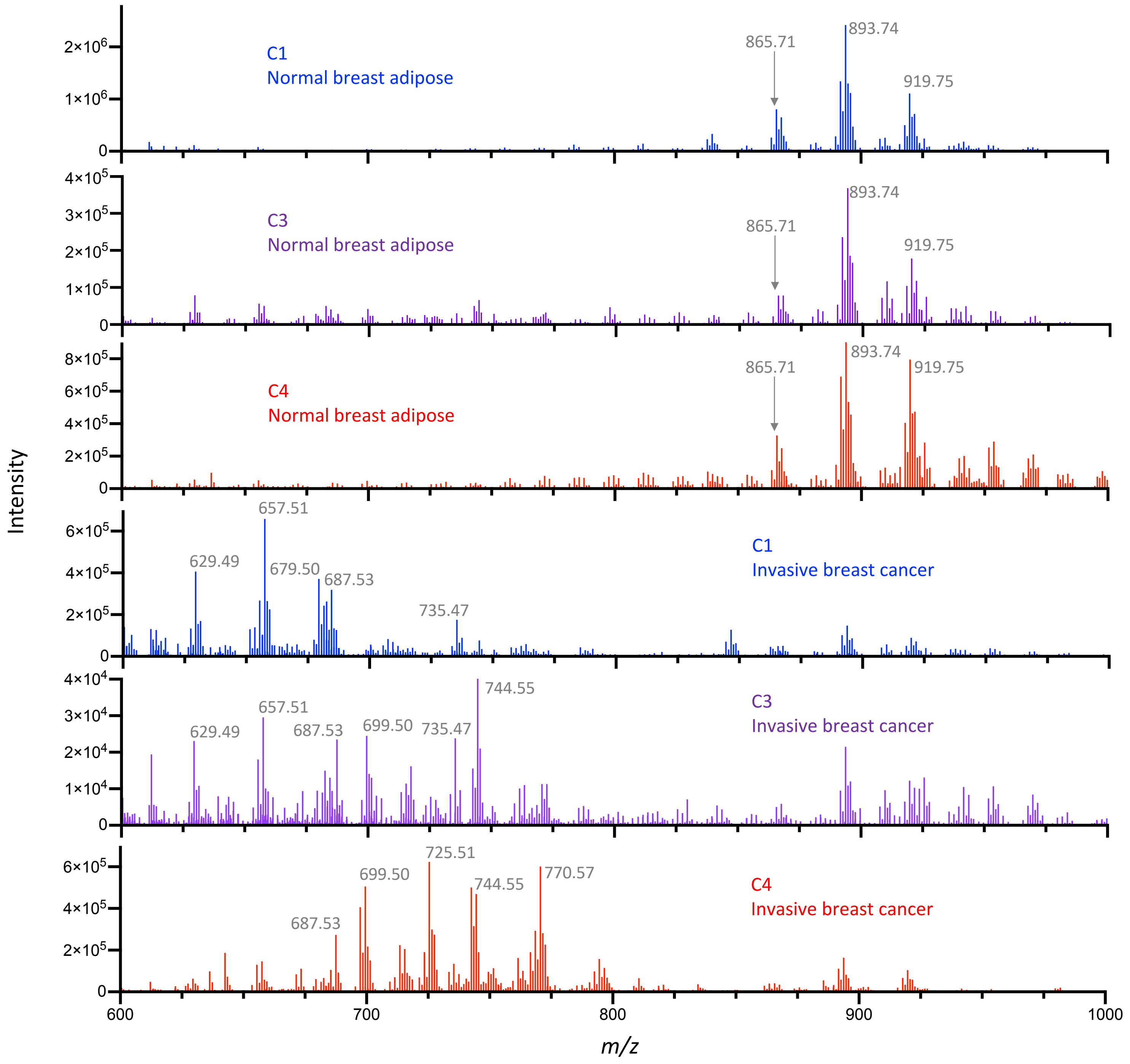

Supplementary Figure S6: REIMS spectra from human breast tissue samples (Coag). Representative REIMS spectra acquired in coagulation mode from either normal adipose or invasive breast cancer samples acquired from patients being treated at the 3 Consortium sites affiliated with clinical centers. The spectra shown are 1s scans acquired using coagulation mode, subjected to background subtraction and lockmass correction. The tissue type was determined by a breast pathologist at each site.

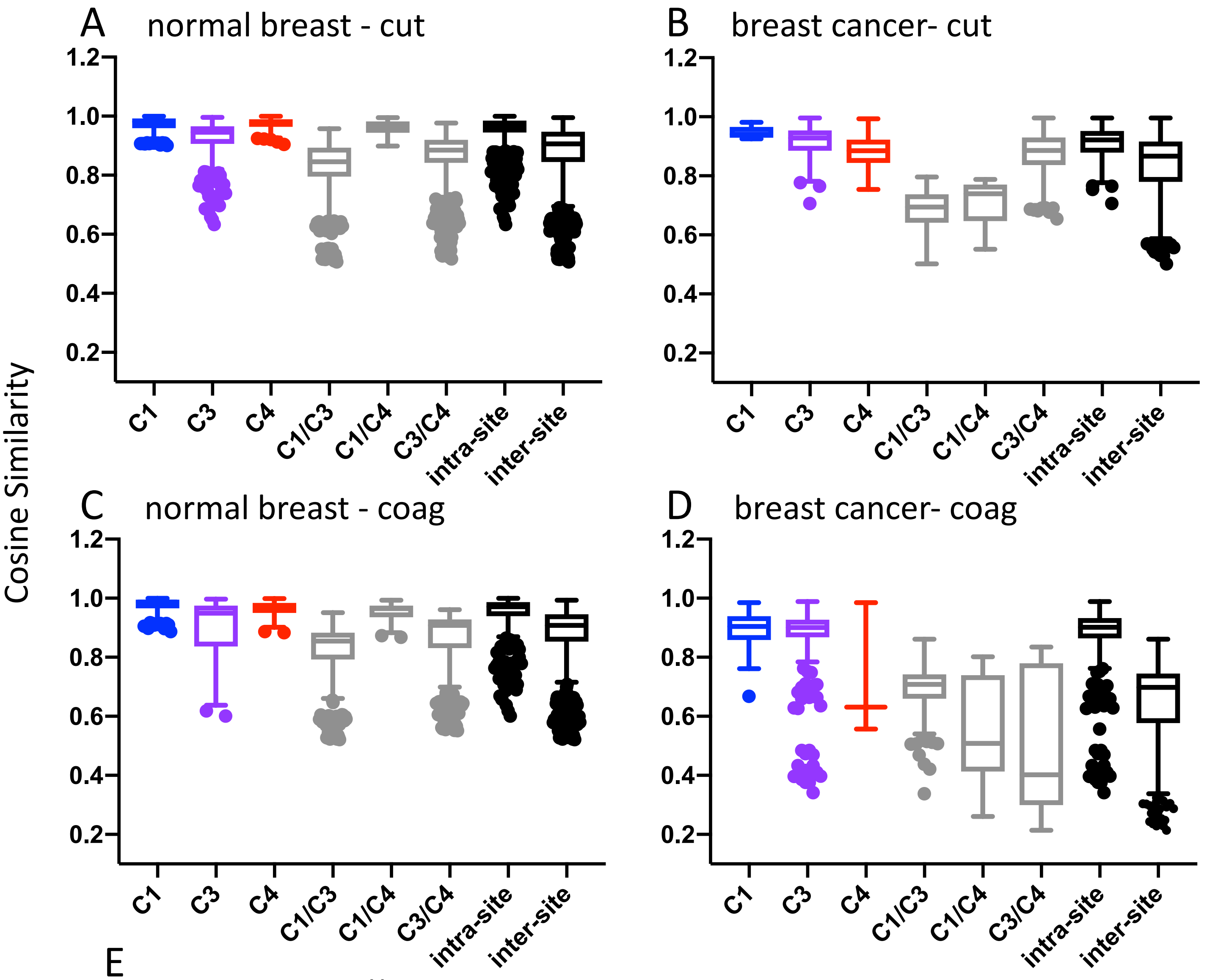

E

Normal breast				Breast cancer			
m/z bin[1] (cut)	Rank[2]	*m/z* bin[1] (coag)	Rank[2]	*m/z* bin[1] (cut)	Rank[2]	*m/z* bin[1] (coag)	Rank[2]
891.72	2	875.77	441	787.55*	276	952.73	562
931.75*	309	867.71	10	981.85*	906	706.55*	872
891.65*	34	916.75*	53	901.78	262	906.75*	3050
913.65*	169	743.52	83	925.78	297	744.45*	1719
893.65*	77	905.70	162	923.76	228	676.45*	2450
929.75*	291	825.55*	189	787.55*	276	967.35*	4291
865.70	14	781.65*	360	679.55*	94	742.54	133
913.75*	200	895.74	5	684.51	86	951.65*	824
915.72	22	769.56	78	927.85*	429	935.35*	4012
868.72	17	891.72	2	756.45	1378	979.73	571

[1] Most abundant *m/z* bin (or corresponding measured mass when a peak was present) from each of 10 groups of correlated bins selected by HSIC LASSO. Absence of a peak is denoted by *.

[2] Rank (bin number) based on relative abundance over 5000 bins (*m/z* 600-1000).

Supplementary Figure S7: Site-based comparison of spectra from clinical samples (*m/z* 600-1000). Specific and overall intra-site and inter-site cosine similarities of spectra from normal breast (A,C) or breast cancer (B,D) are presented. Overall intra-site and inter-site cosine similarities were statistically significant for all tissue types and cautery modes. (E). *M/z* bins (or measured *m/z*) exhibiting the greatest site-based difference are shown, along with their rank by abundance.

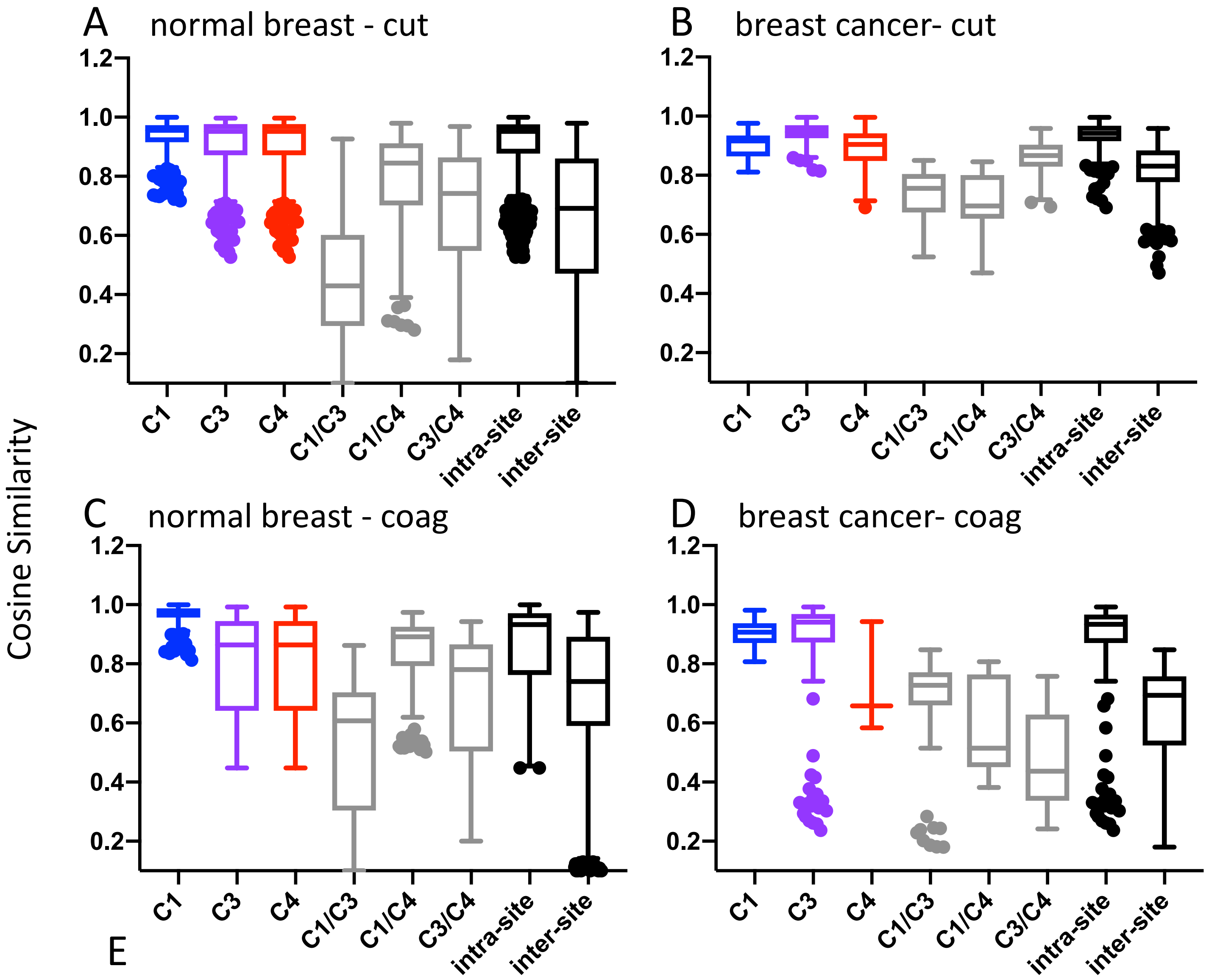

E

Normal breast				Breast cancer			
m/z bin[1] (cut)	Rank	*m/z* bin[1] (coag)	Rank	*m/z* bin[1] (cut)	Rank	*m/z* bin[1] (coag)	Rank
391.22	30	333.25*	431	925.78	1087	112.25*	4025
903.75*	554	867.71	11	956.75*	1865	951.65*	824
929.75*	903	916.75*	68	331.15*	434	675.45*	535
318.15*	458	269.15*	257	163.09	123	315.15*	844
263.18	20	627.55*	852	262.05*	860	952.74	562
891.72	4	808.65*	1203	901.78	976	864.69	1455
915.72	40	913.68	98	279.65*	4187	952.65*	824
924.77	92	599.45*	376	262.05*	860	262.05*	333
973.85*	1760	626.55*	774	254.75*	4524	885.55*	1513
389.15*	109	697.48	119	330.15*	817	469.35*	975

[1] Most abundant *m/z* bin (or corresponding measured mass, when a peak was present) from each of 10 groups of correlated bins selected by HSIC LASSO. Absence of a peak is denoted by (*).
[2] Rank (bin number) based on relative abundance over 9000 bins (*m/z* 100-1000).

Supplementary Figure S8: Site-based comparison of spectra from clinical samples (*m/z* 100-1000). Specific and overall intra-site and inter-site cosine similarities of spectra from normal breast (A,C) or breast cancer (B,D) are presented. Overall intra-site and inter-site cosine similarities were statistically significant for all tissue types and cautery modes. (E). *M/z* bins (or measured *m/z*) exhibiting the greatest site-based difference are shown, along with their rank by abundance.

A normal breast

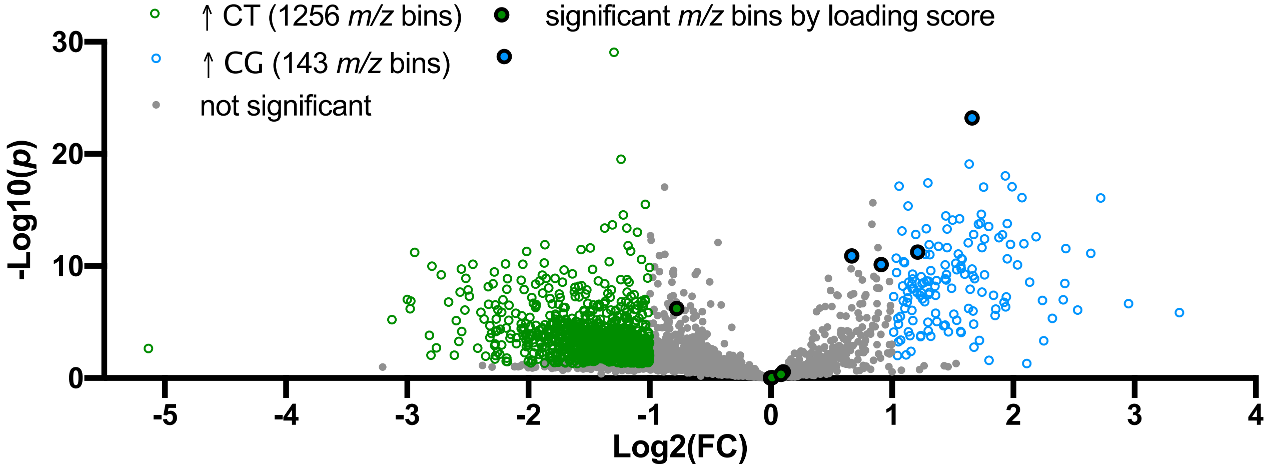

m/z bin[1]	Loading[1]	Log2(FC)	-Log10(*p*)	Rank[2]	*m/z* bin[1]	Loading[1]	Log2(FC)	-Log10(*p*)	Rank[2]
917.74	-0.27	-0.003	0.009	8	909.77	0.20	0.6	11	15
657.55	-0.24	-0.7	6	27	925.72	0.17	1.6	23	21
918.75	-0.20	-0.003	0.008	12	953.75	0.15	1.2	11	30
891.72	-0.18	-0.08	0.33	2	911.78	0.13	0.9	10	32
919.75	-0.16	0.01	0.04	3	895.75	0.12	0.1	0.5	4
744.55[3]	*-0.07*	*-0.7*	*2*	*42*	*893.74[3]*	*0.11*	*0.07*	*0.3*	*1*

B breast cancer

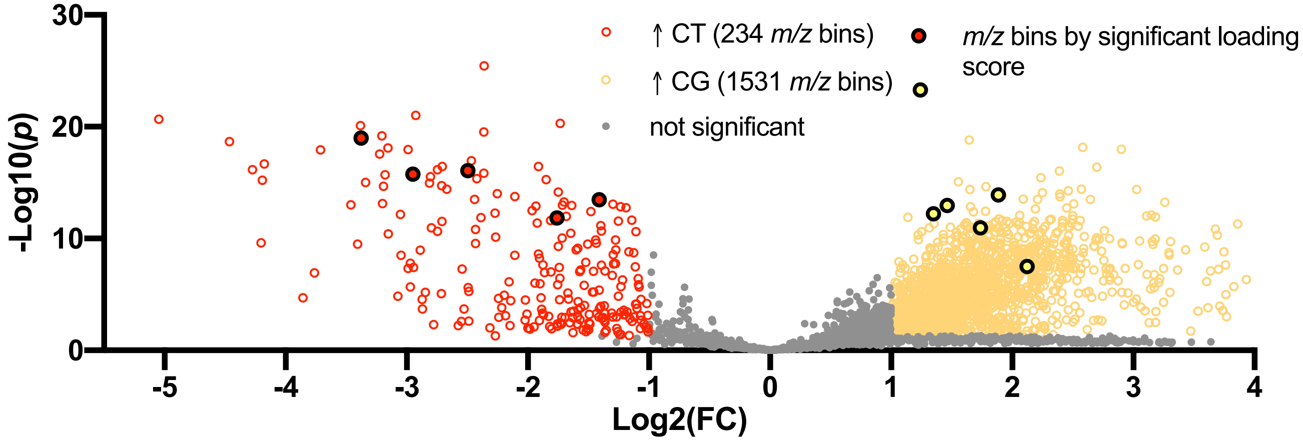

m/z bin[1]	Loading[1]	Log2(FC)	-Log10(*p*)	Rank[2]	*m/z* bin[1]	Loading[1]	Log2(FC)	-Log10(*p*)	Rank[2]
699.50	0.36	-2.9	15	2	657.51	-0.04	1.7	11	1
744.55	0.34	-1.4	13	1	655.50	-0.006	1.9	14	7
687.53	0.21	-1.7	22	3	682.59	-0.043	1.4	12	3
699.55	0.18	-2.5	16	6	656.51	-0.021	1.5	13	10
697.48	0.18	-3.3	19	19	681.52	-0.019	2.1	8	5
744.55[3]	*0.34*	*-1.4*	*13*	*1*	*893.75[3]*	*0.52*	*1.6*	*3*	*2*

[1] *m/z* measured mass selected on the five most significant PCA loading scores
[2] Rank (bin number) based on relative abundance over 5000 bins (*m/z* 600-1000).
[3] For reference, parameters are shown for representative *m/z* bins that differentiate breast cancer from normal breast

Supplementary Figure S9: Differentially-abundant ions in cut as compared with coagulation mode. Volcano plots indicating the number of ions that significantly differ in abundance between cut (CT) and coagulation modes (CG), where $p<0.05$ and $|Log2(FC)|>1$; shown for normal breast (A) or breast cancer tissue (B). *M/z* bins with the most significant PCA loading scores are listed, and indicated on the volcano plot.

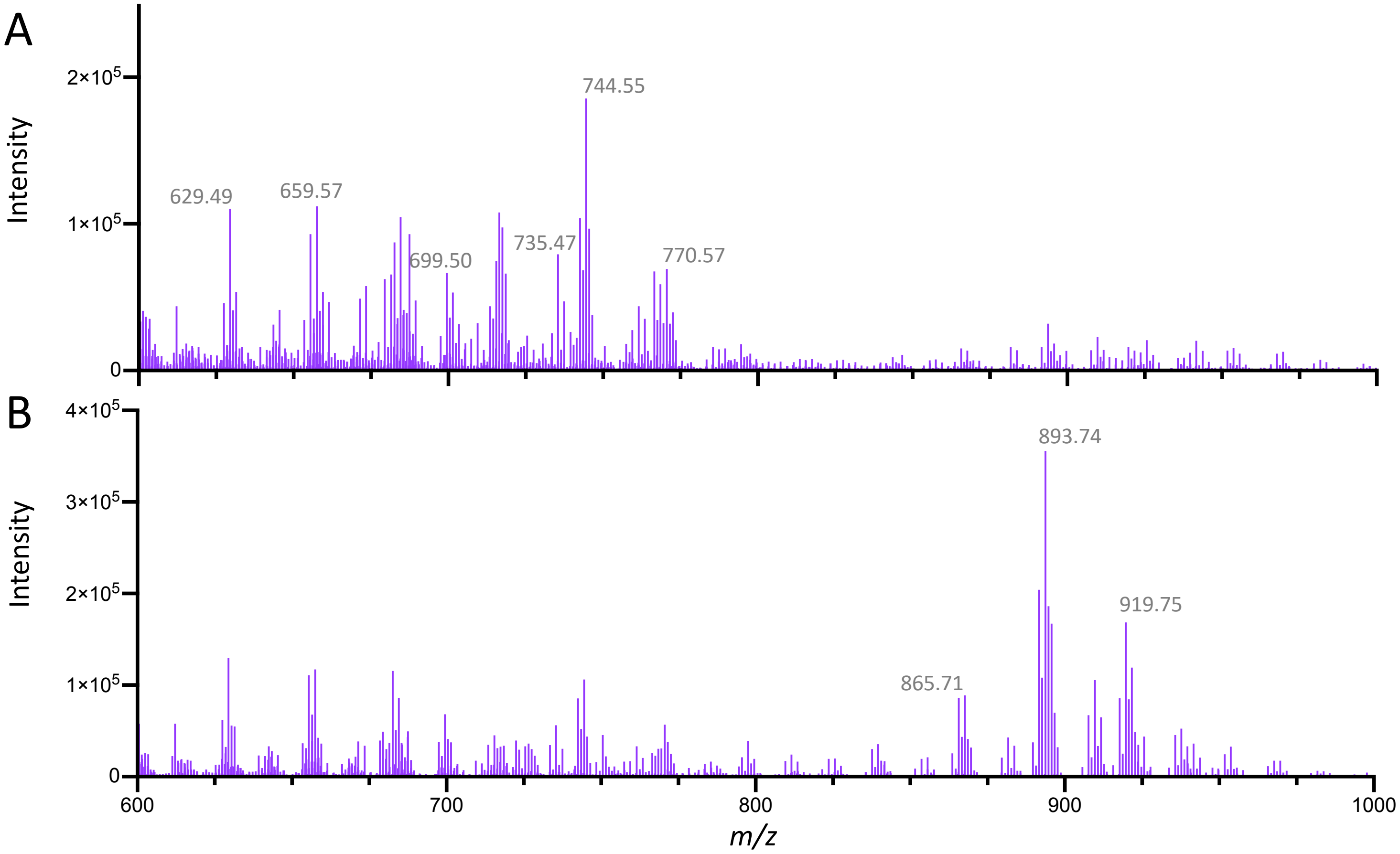

Supplementary Figure S10: Examples of misclassified REIMS spectra. The REIMS spectra above were acquired at C3 in coagulation mode, and were misclassified by the model created using data from C4. The spectrum in A was obtained from pathology-confirmed breast cancer tissue, but was misclassified as ‘normal’ (false negative). The spectrum in B was obtained from pathology-confirmed normal breast tissue but was misclassified as ‘breast cancer’ (false positive).

Table S1: Characterization of selected fatty acids in normal adipose and invasive breast cancer stratified by *PIK3CA* mutation and hormone receptor status

Normal/ Cancer	Abundant in: PIK3CA Wt/Mut	ERPR+/ Triple -	m/z (bin)	m/z (measured)	m/z (theoretical)	m/z (error, ppm)	Putative ID	Ion
Normal	NS[1]	NS	281.25	281.248	281.2486	2.1	FA(18:1)	$[M-H]^-$
Normal	Wt	ERPR+	279.25	279.234	279.2329	3.9	FA(18:2)	$[M-H]^-$
Cancer	NS	NS	307.25	307.264	307.2643	1.0	FA(20:2)	$[M-H]^-$
Cancer	Mut	NS	305.25	305.248	305.2486	2.0	FA(20:3)	$[M-H]^-$
Cancer	NS	NS	303.25	303.233	303.2329	0.3	FA(20:4)	$[M-H]^-$
Cancer	NS	NS	331.25	331.264	331.2643	0.9	FA(22:4)	$[M-H]^-$
Cancer	Mut	NS	329.25	329.249	329.2486	1.2	FA(22:5)	$[M-H]^-$

[1]NS: not significant p>0.05

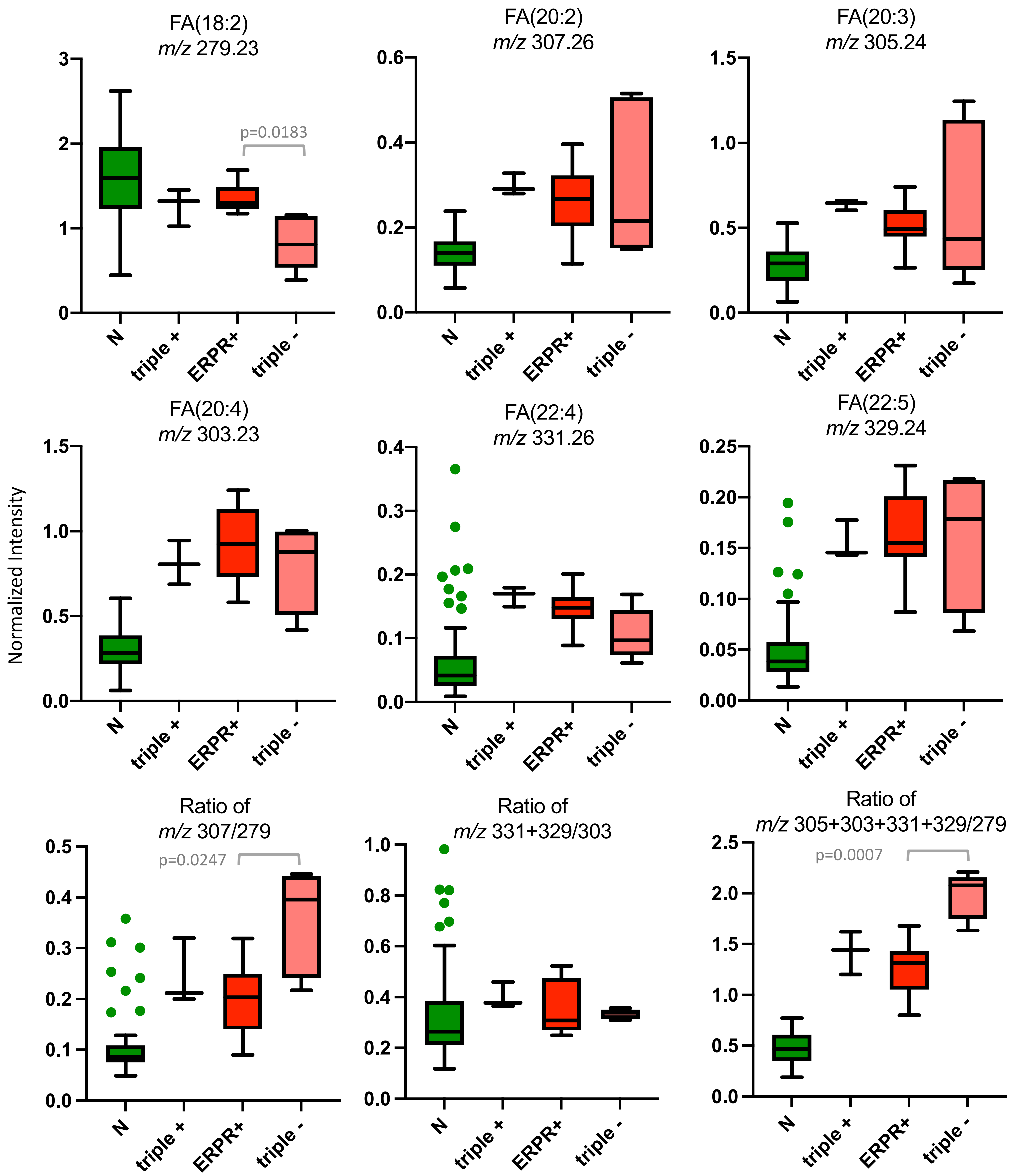

Supplementary Figure S11: Fatty acid metabolism in breast tissue stratified by hormone receptor status. Abundance of fatty acids (or ratios) is shown in triple positive (ER+, PR+,HER2, N=3), ERPR+ (ER+,PR+,HER2-, N=18), and triple – (ER-,PR-,HR-, N=5) breast cancer spectra, as compared with N (normal breast adipose, N=82).
